# Linking Theory to Practice: Predicting Ballistic Performance from Mechanical Properties of Aged Body Armor

**DOI:** 10.6028/jres.125.026

**Published:** 2020-08-24

**Authors:** Amanda L. Forster, Dennis D. Leber, Amy Engelbrecht-Wiggans, Virginie Landais, Allen Chang, Emilien Guigues, Guillaume Messin, Michael A. Riley

**Affiliations:** 1 Material Measurement Laboratory, National Institute of Standards and Technology, Gaithersburg, MD 20899, USA; 2 Information Technology Laboratory, National Institute of Standards and Technology, Gaithersburg, MD 20899, USA; 3 Theiss Research, La Jolla, CA 92037, USA

**Keywords:** artificial aging, ballistic resistance, body armor, field aging

## Abstract

It has long been a goal of the body armor testing community to establish an individualized, scientific-based protocol for predicting the ballistic performance end of life for fielded body armor. A major obstacle in achieving this goal is the test methods used to ascertain ballistic performance, which are destructive in nature and require large sample sizes. In this work, using both the Cunniff and Phoenix-Porwal models, we derived two separate but similar theoretical relationships between the observed degradation in mechanical properties of aged body armor and its decreased ballistic performance. We present two studies used to validate the derived functions. The first correlates the degradation in mechanical properties of fielded body armor to the degradation produced by a laboratory accelerated-aging protocol. The second examines the ballistic resistance and the extracted-yarn mechanical properties of new and laboratory-aged body armor made from poly(p-phenylene-2,6-benzobisoxazole), or PBO, and poly(p-phenylene terephthalamide), or PPTA. We present correlations found between the tensile strengths of yarns extracted from armor and the ballistic limit (V50) when significant degradation of the mechanical properties of the extracted yarns was observed. These studies provided the basis for a validation data set in which we compared the experimentally measured V50 ballistic limit results to the theoretically predicted V50 results. The theoretical estimates were generally shown to provide a conservative prediction of the ballistic performance of the armor. This approach is promising for the development of a tool for fielded armor performance surveillance relying upon mechanical testing of armor coupon samples.

## 1 Introduction

In the wake of a 2003 field failure of a piece of body armor composed of poly(*p*-phenylene-2,6-benzobisoxazole) [1–3], or PBO, questions have been raised regarding the expected service life of ballistic-resistant body armor. A study conducted prior to 1990 on body armor made from poly(*p*-phenylene terephthalamide), or PPTA, indicated that armor that had been in use for approximately 10 years could still defeat the threats it was designed to stop [4]. The conclusion in this 1986-published study is no longer applicable because the armor used in that study is not representative of the materials and construction techniques that are used in modern body armor. For the last decade, the body armor community has conducted major research efforts to understand the effects of field and laboratory aging on the performance of body armor [5–7]. These efforts culminated in a revised standard [8] for ballistic-resistant body armor that included an environmental conditioning protocol to test the capability of a given armor design to withstand conditions of heat, moisture, and mechanical wear, which may translate to enhanced confidence in the long-term performance of the armor.

To assess the long-term field performance of armor, an ideal study would consist of issuing a single body armor design to thousands of end users who work in an array of different types of positions (e.g., patrol officers vs. detectives) in a diversity of climates (e.g., southern United States vs. northern Canada). Armor would then be removed from service for analysis at specified intervals. Unfortunately, this ideal study has proven difficult to execute in practice because no single design of body armor has ever been issued to a large number of end users. In reality, law enforcement officers in the United States and across the world utilize a variety of different armor designs. Furthermore, the actual use condition for an individual piece of armor is incredibly challenging to precisely assess: Officers can be reassigned or promoted over the course of a few years of service, thus changing the use of their armor; some officers do not wear their armor regularly; and some officers may not follow common standards of care for their armor (e.g., some may hang it when it is not being worn, while others may leave it in the bottom of their locker or the trunk of their car until it is needed again).

A study of field-aged armor was conducted in Canada [9, 10]; however, interpretations of the data were complicated because of the wide variety of armor designs sampled. The study was further complicated by the difficulty inherent in assessing the performance of the armor at the end of its life cycle without a consistent and accurate benchmark for the performance of that armor when it was new. The benchmark used was of questionable accuracy due to the limited number and placement of shots required at the time to assess the armor performance when it was new, as specified by previous versions of the National Institute of Justice (NIJ) body armor standard to which the armor was originally certified, but since revised, as is discussed later herein. These issues, and the variability that resulted in benchmark values, led the authors of the Canadian study to conclude that correlations could not be established between armor age and ballistic performance.

Most armor sold in the United States for use by law enforcement officers has a 5 year warranty period. Typically, one would expect body armor to be designed with a “safety margin” to account for the potential of a reduction in armor performance over time due to use and wear [1–3]. Because of the aforementioned variations in armor design, use, and care, such a warranty period and expected safety margin cannot be uniformly assumed to be analogous with “safe for use” across the population of fielded armor. This individuality of fielded body armor presents substantial challenges for organizations in defining effective armor surveillance and replacement policies. While performing a large-scale study to examine fielded armor performance remains challenging, we attempted herein to combine available data from fielded and laboratory-aged armor to better understand armor long-term performance, where the focus was on correlating the measured tensile strength of component yarns to the armor’s measured ballistic limit (*V*_50_), whether new or aged.

The *V*_50_ is defined by ASTM Standard E3110-18 as “the velocity at which 50% of the impacts by a specified test threat are expected to completely penetrate nominally identical test items when tested according to a specified test method” [11]. The *V*_50_ provides a convenient mechanism to track relative changes in ballistic performance that might not be revealed by a perforation test, which only investigates the ability of an armor to stop threats of a specified velocity.

**Fig. 1:**
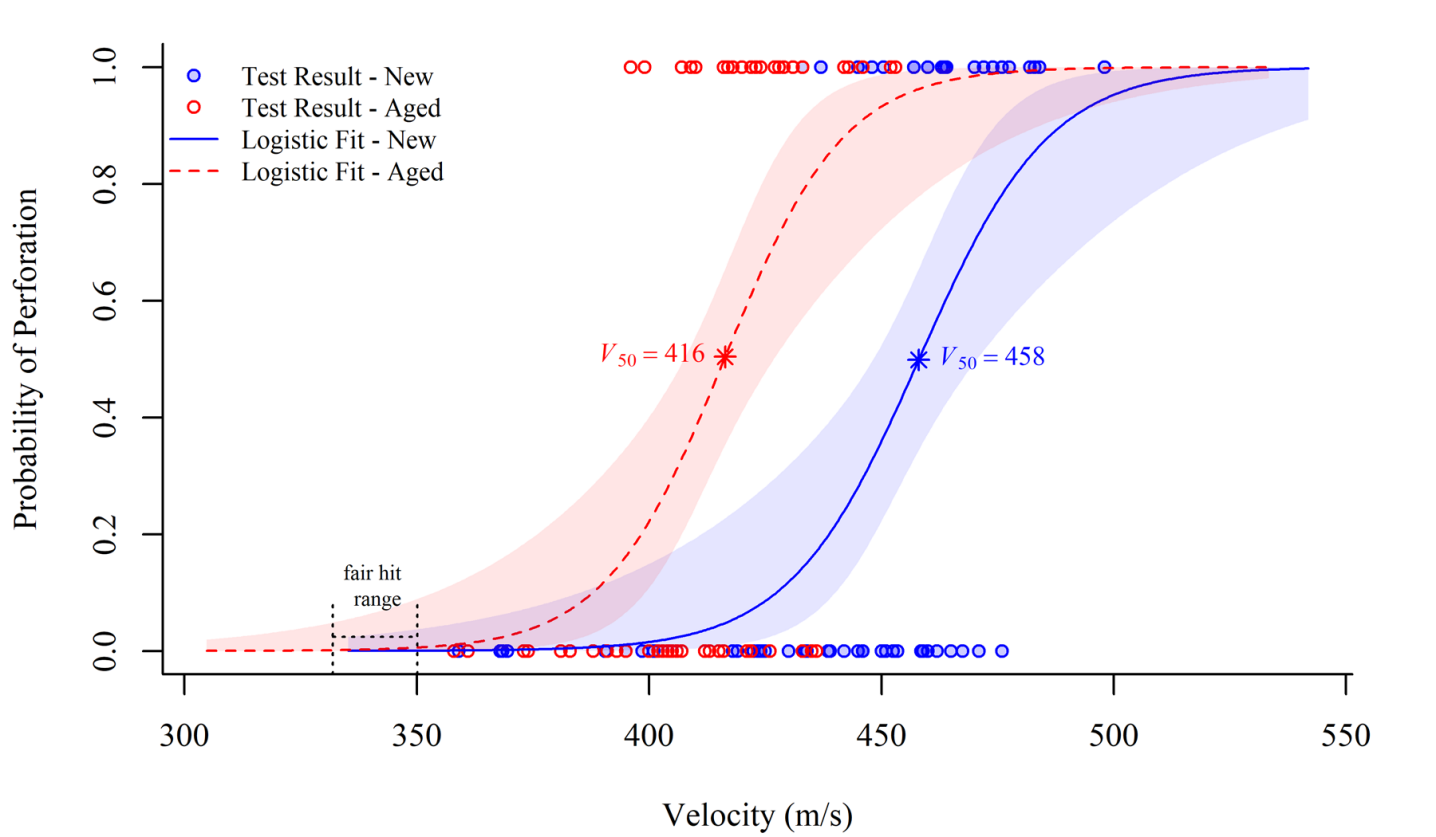
Logistic regression models for the probability of perforation for new and aged PBO body armor. The circles represent the threat velocity and perforation result (1 = perforation, 0 = no perforation) of the V_50_ test. The shaded regions are the 95% confidence intervals of the logistic regression fit.

Using a logistic regression model to describe the probability of perforation, Fig. 1 illustrates the ballistic performance differences between new and aged armor. The details of the application of the logistic regression analysis to *V*_50_ data are reported elsewhere [8, 12]. Figure 1 shows the probability-of-perforation curves for a new PBO armor and for a PBO armor of the same model after being artificially aged using the conditioning protocol described in NIJ Standard 0101.06 [8].

The NIJ Standard 0101.04 [13] threats were used in this investigation because they matched the conditions of a known field failure for PBO body armor. This standard defines a “fair hit” velocity, for a 9 mm, 8.04 g full-metal-jacket projectile for the “fair hit” range, as a shot with a velocity of 341 m/s ± 9.1 m/s. This fair hit range is indicated in Fig. 1. We note that the estimated probability of perforation at the upper bound of the fair hit velocity interval (350.1 m/s) for the new armor is about 0.04%, with an upper 95% confidence bound of 3.7%. The estimated *V*_50_ provided by the logistic model for the new armor is 458 m/s, which exceeds the upper bound of the fair hit velocity interval, 350.1 m/s, by about 31%. In contrast, for the aged armor, the estimated probability of perforation at the upper bound of the fair hit velocity interval is approximately 0.6%, with an upper 95% confidence bound of 8.9%. Furthermore, the estimated *V*_50_ for the aged armor, which is 416 m/s, exceeds the upper bound, 350.1 m/s, of the fair hit velocity interval by about 19%. Both the downward shift in *V*_50_ and the upward shift in the probability of perforation at the upper bound of the fair hit velocity interval for aged armor, as compared to new armor, indicate that the ballistic performance of the armor has declined with aging. This example signifies the importance of investigating the effect of aging on armor systems.

Beyond the logistic regression modeling of empirical *V*_50_ test results, there are theoretical methods for considering the influence of material properties on ballistic performance [14–19]. These methods focus on the concepts of strain-wave speed and specific toughness. The strain-wave speed represents the speed of energy dissipation away from a point of ballistic impact, and the specific toughness represents the approximate amount of elastic energy a fiber can withstand or absorb before failure, as normalized by mass. In an effort to combine these concepts into one term, which can be expressed as a ballistic property (a theoretical *V*_50_), Cunniff determined a relationship between dimensionless parameters based on extensive experimental data [15]. Phoenix and Porwal also related the concepts of strain wave, specific toughness, and *V*_50_ and developed a theoretical membrane model for an in-plane isotropic material [16–18].

In the following sections, we leveraged the works of Cunniff and Phoenix-Porwal to derive two separate, but similar theoretical models that relate a decrease in the material properties of yarns used in armor to a decrease in the armor’s *V*_50_. We then describe a study that links the mechanical properties of artificially aged armor with those of field-aged armor. This is followed by further study of the artificially aged armor wherein a positive correlation between tensile strength of yarns extracted from the armor and the *V*_50_ ballistic limit was observed. This work formed the basis for the data set used in the validation of our derived models. We conclude with suggestions on how these results might facilitate an individualized armor surveillance program.

## 2 Derivation of Theoretical Degradation Relationships

The results of different theoretical studies agree in that the concepts of specific toughness and strain-wave speed are critical in predicting ballistic performance [14–16, 20]. The strain-wave speed is the speed of energy dissipation away from a point of ballistic impact and is calculated as the square root of the ratio of a fiber’s Young’s modulus, *E*, to the fiber density, ρ: $\sqrt{\frac{E}{\rho }}$. Specific toughness is the approximate amount of stored elastic energy a fiber can withstand before failure, as normalized by mass density, and it is calculated as the ratio of the product of fiber ultimate axial tensile strength, σ, and fiber ultimate tensile strain, ε, over twice fiber density: $\frac{\sigma \varepsilon }{\left(2\rho \right)}$. The mechanical properties of a fiber can be readily determined from tensile testing, so the relationships between fiber properties and ballistic performance can, in theory, allow for prediction of a material’s ballistic performance based on simple tests. Full details of this analysis are given in another publication [21], and a brief summary is also included below.

### 2.1 Cunniff’s Tensile-Strength-to-*V*_50_ Relation

Cunniff’s paper [15] examined a large base of experimental data, aiming to determine relationships between material properties and *V*_50_ that hold for different materials. The variables of interest that he identified were the fiber toughness, fiber strain-wave velocity, *V*_50_, and a ratio, $\Gamma_{0}$, of projectile and system areal density,

$\Gamma_{0}=\frac{A_{d}A_{p}}{m_{p}}$, (1)

where *A_d_* is the armor system areal density, *A_p_* is the projectile presented area to the target, and *m_p_* is the projectile mass. He then used dimensional analysis of the experimental *V*_50_ data obtained over a wide range of parameter values (including projectile and target dimensions and masses and fiber mechanical properties and densities) to determine a function, Φ, between two dimensionless parameters that resolved and fit the experimental data for all but one of the material systems of interest, namely:

$\Phi \left(\Gamma_{0},\frac{V_{50}}{\Omega^{\frac{1}{3}}}\right)=0$, (2)

where Ω is the product of the material’s specific toughness with the strain-wave velocity:

$\Omega =\frac{\sigma \varepsilon }{2\rho }\sqrt{\frac{E}{\rho }}$ . (3)

Aging the material can cause changes in its mechanical properties, such as ultimate tensile strength, ultimate tensile strain, and Young’s modulus, but the material density is assumed to stay constant. (This assumption was verified in Ref. [22], where density was found to be independent of water sorption/desorption.) The ratio of the *V*_50_ of aged material to that of unaged material, for a constant projectile type, can then be calculated as a function of these material parameters as follows: For fixed projectile type, the areal density ratio, $\Gamma_{0}$, is constant, so the second dimensionless term in Eq. (2) must also remain constant because Eq. (2) is bijective. Thus, *V*_50_ is proportional to the cubed root of Ω:

$V_{50\;C}\propto \Omega^{\frac{1}{3}}$, (4)

where the “C” subscript refers to the Cunniff model. Writing this for both aged and unaged material and taking the ratio yields the percent retention of *V*_50_ for the Cunniff model, $rV_{50\;C}$,

$rV_{\text{50}\;C}=\frac{V_{50\;C\;\text{aged}}}{V_{50\;C\;\text{new}}}\times 100={\left(\frac{\Omega_{\text{aged}}}{\Omega_{\text{new}}}\right)}^{\frac{1}{3}}\times 100={\left(\frac{\sigma_{\text{aged}}\varepsilon_{\text{aged}}}{2\rho }\sqrt{\frac{E_{\text{aged}}}{\rho }}\frac{2\rho }{\sigma_{\text{new}}\varepsilon_{\text{new}}}\sqrt{\frac{\rho }{E_{\text{new}}}}\right)}^{\frac{1}{3}}\times 100\;\;\;\;\;={\left(\frac{\sigma_{\text{aged}}\varepsilon_{\text{aged}}}{\sigma_{\text{new}}\varepsilon_{\text{new}}}\sqrt{\frac{E_{\text{aged}}}{E_{\text{new}}}}\right)}^{\frac{1}{3}}\times 100$. (5)

Ballistic materials typically exhibit a linear stress strain curve without any yielding or other nonlinearities, such that

$E=\frac{\sigma }{\varepsilon }$. (6)

Substituting Eq. (6) into Eq. (5) gives the expression

$rV_{\text{50}\;C}=\frac{V_{\text{50}\;C\;\text{aged}}}{V_{\text{50}\;C\;\text{new}}}\times 100={\left(\frac{\sigma_{\text{aged}}\varepsilon_{\text{aged}}}{\sigma_{\text{new}}\varepsilon_{\text{new}}}\sqrt{\frac{\sigma_{\text{aged}}\varepsilon_{\text{new}}}{\sigma_{\text{new}}\varepsilon_{\text{aged}}}}\right)}^{\frac{1}{3}}\times 100={\left(\frac{\sigma_{\text{aged}}}{\sigma_{\text{new}}}\right)}^{\frac{1}{2}}{\left(\frac{\varepsilon_{\text{aged}}}{\varepsilon_{\text{new}}}\right)}^{\frac{1}{6}}\times 100$. (7)

From Eq. (7), the $rV_{50\;C}$ of a material after aging can be determined as a simple function of the aged and unaged ultimate stress and strain.

### 2.2 Phoenix-Porwal’s Tensile-Strength-to-*V*_50_ Relation

Cunniff derived Eq. (2) and Eq. (3) empirically from extensive data [15]. Taking a theoretical approach, Phoenix and Porwal modeled ballistic impact into a homogeneous, in-plane, isotropic membrane [17, 23]. Their analysis made some assumptions that are inaccurate for ballistic materials; however, their result is surprisingly similar to Cunniff’s experimentally determined result. Phoenix and Porwal derived the following relation,

$V_{\text{50 PP}}=\Omega^{\frac{1}{3}}\frac{2^{\frac{1}{3}}\varepsilon^{\frac{1}{12}}\left(1+\theta^{2}\Gamma_{0}\right)}{K_{max}^{\frac{3}{4}}}$, (8)

where Ω and $\Gamma_{0}$ are the same as the variables given in the Cunniff’s model, in Eq. (1) and Eq. (3), respectively, θ is an adjustment parameter, typically between 1 and 2, to account for various factors such as plastic projectile nose deformation, fabric wraparound, etc., and $K_{max}$ is given by:

$K_{\max}=\exp\left\{-\frac{4\;\;\theta^{2}\;\Gamma_{0}\left(\psi_{\max}^{2}-1\right)}{3\left(1+\theta^{2}\;\Gamma_{0}\right)}\right\}\psi_{\max}^{\frac{1}{2}}{\left[\frac{\sqrt{\frac{\psi_{\max}}{\varepsilon }}\left(\psi_{\max}-1\right)}{\ln\left\{1+\sqrt{\frac{\psi_{\max}}{\varepsilon }}\left(\psi_{\max}-1\right)\right\}}\right]}^{\frac{2}{3}}$, (9)

where $\psi_{max}$ is approximated by

$\psi_{max}\approx \sqrt{\frac{1+\theta^{2}\Gamma_{0}}{2\theta^{2}\Gamma_{0}}}$ . (10)

As above, to predict *V*_50_ reduction after aging, the projectile-dependent parameters θ, $\Gamma_{0}$, and $\psi_{max}$ can be held constant, such that

$V_{50}\propto \Omega^{\frac{1}{3}}\varepsilon^{\frac{1}{12}}{\left[\frac{\ln\left\{1+\sqrt{\frac{\psi_{\max}}{\varepsilon }}\left(\psi_{\max}-1\right)\right\}}{\sqrt{\frac{\psi_{\max}}{\varepsilon }}\left(\psi_{\max}-1\right)}\right]}^{\frac{1}{2}}$. (11)

Thus, the retention of *V*_50_ according to the Phoenix-Porwal model, $rV_{\text{50}\;\text{PP}}$, is obtained as follows, when using Eq. (8) through Eq. (11):

$rV_{\text{50}\;\text{PP}}=\frac{V_{\text{50}\;\text{PP aged}}}{V_{\text{50}\;\text{PP new}}}\times 100={\left(\frac{\Omega_{\text{aged}}}{\Omega_{\text{new}}}\right)}^{\frac{1}{3}}{\left(\frac{\varepsilon_{\text{aged}}}{\varepsilon_{\text{new}}}\right)}^{\frac{1}{12}}{\left(\frac{K_{\text{max new}}}{K_{\text{max aged}}}\right)}^{\frac{3}{4}}\times 100$

$\;\;\;\;\;={\left(\frac{\sigma_{\text{aged}}\;}{\sigma_{\text{new}}\;}\right)}^{\frac{1}{2}}{\left(\frac{\varepsilon_{\text{aged}}}{\varepsilon_{\text{new}}}\right)}^{\frac{1}{6}}{\left(\frac{\varepsilon_{\text{aged}}}{\varepsilon_{\text{new}}}\right)}^{\frac{1}{12}}{\left(\sqrt{\frac{\varepsilon_{\text{aged}}}{\varepsilon_{\text{new}}}}\frac{\ln\left\{1+\sqrt{\frac{\psi_{\max}}{\varepsilon_{\text{aged}}}}\left(\psi_{\max}-1\right)\right\}}{\ln\left\{1+\sqrt{\frac{\psi_{\max}}{\varepsilon_{\text{new}}}}\left(\psi_{\max}-1\right)\right\}}\right)}^{\frac{1}{2}}\times 100$, (12)

$\;\;\;\;\;={\left(\frac{\sigma_{\text{aged}}\;}{\sigma_{\text{new}}\;}\frac{\varepsilon_{\text{aged}}}{\varepsilon_{\text{new}}}\frac{\ln\left\{1+\sqrt{\frac{\psi_{\max}}{\varepsilon_{\text{aged}}}}\left(\psi_{\max}-1\right)\right\}}{\ln\left\{1+\sqrt{\frac{\psi_{\max}}{\varepsilon_{\text{new}}}}\left(\psi_{\max}-1\right)\right\}}\right)}^{\frac{1}{2}}\times 100$

where the $rV_{\text{50}\;\text{PP}}$ of a material after aging is a function of the aged and unaged ultimate stress and strain.

### 2.3 Comparing the Cunniff and Phoenix-Porwal Models

While the Phoenix-Porwal model’s *V*_50_ retention equation, Eq. (12), is more complicated than the Cunniff model’s corresponding equation, Eq. (7), the two analyses give remarkably similar results. Taking Eq. (12) and dividing by Eq. (7) gives the factor that differs between these two equations:

$\frac{rV_{\text{50}\;\text{PP}}}{rV_{\text{50}\;C}}={\left(\frac{\varepsilon_{aged}}{\varepsilon_{new}}\right)}^{\frac{1}{3}}{\left[\frac{\ln\left\{1+\sqrt{\frac{\psi_{\max}}{\varepsilon_{aged}}}\left(\psi_{\max}-1\right)\right\}}{\ln\left\{1+\sqrt{\frac{\psi_{\max}}{\varepsilon_{new}}}\left(\psi_{\max}-1\right)\right\}}\right]}^{\frac{1}{2}}$. (13)

Furthermore, it can be shown that if $\varepsilon_{\text{aged}}<\varepsilon_{\text{new}}$, then the *V*_50_ retention as predicted by the Phoenix-Porwal model in Eq. (12) will be more conservative, i.e., lower, than that predicted by the Cunniff model in Eq. (7).

To enumerate, the full-metal-jacket round nose bullets that were used in this study had nominal masses of 8 g, and their nominal projectile presented area to the target was *A_p_* = 6.36 × 10^−5^ m^2^. The PPTA and PBO armors had areal densities of 2.625 kg/m^2^ and 2.273 kg/m^2^, respectively, resulting in $\Gamma_{0}$ values of 0.0209 and 0.0181, as shown in Table 1. Table 2 presents values for the ratio in Eq. (13) for the typical $\psi_{max}$ values presented in Table 1.

**Table 1:** Typical areal density and $\psi_{max}$values at the indicated θ value for materials used in this study.

		PPTA	PBO
*A_d_* (kg/m^2^)		2.63	2.27
*A_p_* (m^2^)		6.36E-05	6.36E-05
*m_p_* (g)		8.0	8.0
$\Gamma_{0}$		0.0209	0.0181
$\psi_{max}$	θ=1.25	3.98	4.27
	θ=1.5	3.34	3.58
	θ=1.75	2.88	3.09
	θ=2	2.55	2.72

**Table 2:** Typical values of Eq. (13) when $\varepsilon_{\text{new}}=0.03$.

$\varepsilon_{\text{aged}}$	$\psi_{max}$= 2.5	$\psi_{max}$= 3	$\psi_{max}$= 3.5	$\psi_{max}$= 4	$\psi_{max}$= 4.5
0.10	1.33	1.35	1.36	1.37	1.37
0.05	1.13	1.14	1.14	1.14	1.15
0.04	1.07	1.08	1.08	1.08	1.08
0.03	1.00	1.00	1.00	1.00	1.00
0.02	0.90	0.90	0.90	0.90	0.90
0.01	0.76	0.75	0.75	0.74	0.74

### 2.4 Summary

Both Eq. (7) and Eq. (12) relate changes in a material’s tensile properties to changes in ballistic properties. These equations were separately derived from theory and empirical testing, yet the ratio in Eq. (13) is around 0.9 to 1.1 (as seen in Table 2), so these models give very similar results. These relations between tensile and ballistic properties allow the ballistic performance of a material to be predicted as the material degrades by performing tensile tests, which require less material and are less costly than ballistic testing.

## 3 Validation Data: Mechanical Properties and *V*_50_ Ballistic Performance

To validate the derived theoretical relationships between mechanical properties and ballistic performance, we leveraged data from two different studies. We first examined the effect of degraded material properties on ballistic performance in armor that was subjected to artificial aging protocols within the laboratory. Then, we investigated the relationship between the degradation of material properties in artificially aged laboratory testing to the material properties found in previously fielded armor. All data associated with this publication have been archived at https://doi.org/10.18434/M32179.

### 3.1 Linking Extracted Yarn Tensile Strength to *V*_50_

To support the development of a revised NIJ body armor standard [8], armor constructed from two different materials (PBO and PPTA) were subjected to laboratory artificial-aging protocols. Mechanical properties of yarns extracted from these artificially aged vests were obtained, as were ballistic performance measures in the form of *V*_50_ estimates. In the following sections, we describe the armor, aging, and testing protocols used in the study. We conclude by providing a summary of the analysis and conclusions that relate the degradation in the mechanical properties of extracted yarns to a decrease in ballistic performance.

#### 3.1.1 Armor Description

Two different types of armors were examined herein. One sample armor was constructed of 20 layers of plain woven fabric made from 0.055 g/m (500 denier) PBO yarns, with spacings of 1.02 yarns per mm (26 yarns per inch) in both the horizontal and vertical directions (as extracted from the armor). The layers of fabric were stitched together in two packs of 10 layers each with a 2.54 cm diagonal quilt stitch to form the ballistic package. This ballistic package was then encased in a stitched moisture-permeable fabric cover and inserted into a lightweight polycotton carrier to form an armor panel. This construction was nearly identical to the one that had failed in the field [1, 2, 24].

The other armor was constructed of 25 layers of plain woven 0.055 g/m (500 denier) PPTA, with 0.945 yarns per mm (24 yarns per inch) in both the horizontal and vertical directions. The layers of fabric were stitched together in one package with a 3.18 cm diagonal quilt stitch to form the ballistic package. This ballistic package was then encased in a water-repellent-treated nylon fabric cover and inserted into a medium-weight polycotton carrier to form an armor panel. This armor was selected because it was known to have good long-term field performance. All armors were manufactured specifically for the study. The PBO armor samples were designed to be NIJ Standard 0101.04 Level IIA compliant, and PPTA armor samples were designed to be NIJ Standard 0101.04 Level II compliant [13] . Both armor samples were constructed to be the size required for NIJ Standard 0101.04 2005 Interim Requirements [25] compliance testing.

#### 3.1.2 Artificial Aging

To accelerate the degradation of mechanical properties in the laboratory, the sample armors were simultaneously exposed to elevated temperature, elevated humidity, and mechanical stress through tumbling. The temperature and humidity were held constant throughout the duration of the aging process. Different combinations of temperature, relative humidity (RH), and duration settings were applied to several different test lots (groups) of sample armors. Temperature and relative humidity settings ranged from laboratory ambient conditions (22 °C and 51% RH) to 70 °C and 90% RH, and duration settings ranged from 10 d to 13 d. In all cases, the sample armors were tumbled at 0.083 Hz (5 rpm) inside a tumbler that met the specifications of NIJ Standard 0101.06 [8]. The specific aging conditions for each armor lot are provided in Table 3, and further details of the aging conditions can be found in a previous publication [6].

**Table 3:** Artificial aging conditions.

**Lot**	Armor Material	Aging Conditions			
		Temp (°C)	Humidity (% RH)	Tumbling (Hz)	Duration (d)
**A1**	PPTA	- - - New armor: no aging - - -			
**A2**	PPTA	22	51	0.083	13
**A3**	PPTA	65	80	0.083	10
**A4**	PPTA	70	90	0.083	13
**B1**	PBO	- - - New armor: no aging - - -			
**B2**	PBO	22	51	0.083	13
**B3**	PBO	65	80	0.083	10
**B4**	PBO	70	90	0.083	13

#### 3.1.3 Determination of Extracted Yarn Mechanical Properties

To obtain yarn mechanical properties, tensile testing of yarns extracted from the woven fabric inside of the armor was carried out in accordance with ASTM D2256-02 [26], “Standard Test Method for Tensile Properties of Yarn by the Single-Strand Method.” Ten to fifteen yarn specimens from the armors of interest were extracted, and the tensile strength at failure was determined. Testing was performed using a universal test frame equipped with a 91 kg load cell, and pneumatic yarn and cord grips (Instron^1^1 Certain commercial equipment, instruments, software, or materials are identified in this paper to provide a full description of the procedures used. Such identification does not imply recommendation or endorsement by the National Institute of Standards and Technology, nor does it imply that the materials or equipment identified are necessarily the best available for the purpose. model 2714-006). The jaw separation was nominally 7.9 cm, and the cross-head speed was approximately 2.3 cm/min. The extracted yarns were nominally 41 cm long and were given 64 twists (1.57 turns/cm or 4 tpi) on a custom-designed yarn-twisting device. This level of twist was maintained on the yarns as they were inserted into the pneumatic yarn and cord grips. Strain measurements were made with a noncontacting video extensometer in conjunction with black foam markers placed approximately 2.5 cm apart in the gauge section of the yarn.

#### 3.1.4 Ballistic Testing

The *V*_50_ was used as a measure of the armor’s ballistic performance. The *V*_50_ measurements were executed according to NIJ Standard 0101.04. Because the mechanical properties and the ballistic testing were destructive, no single armor panel could be subjected to both types of measurements. *V*_50_ measurements were observed from at least one armor panel from each of the test lots with the exception of lot B4, where no *V*_50_ ballistic data were obtained due to sample constraints.

#### 3.1.5 Results and Discussion

**Fig. 2:**
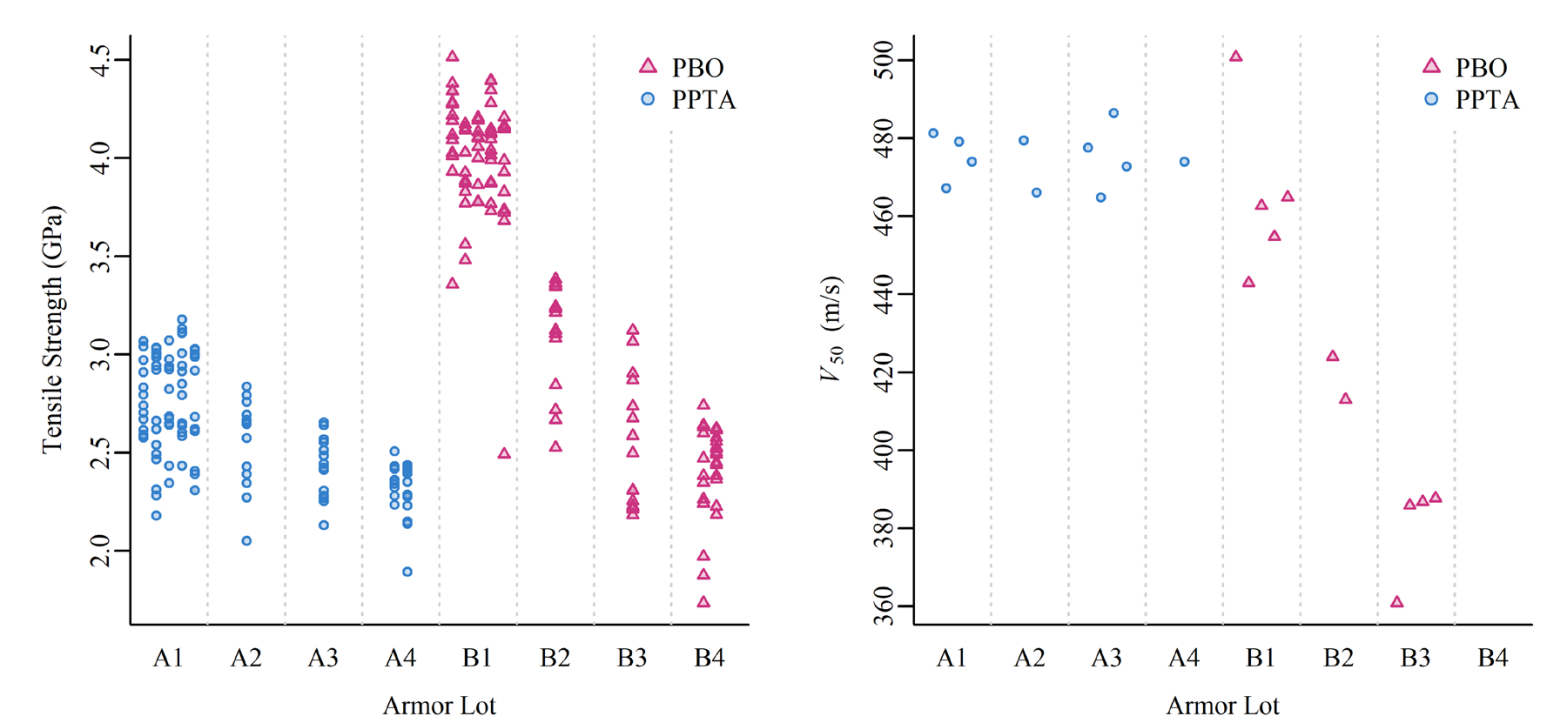
Left panel: Tensile strength measurements, where multiple fibers were measured from an individual vest to create the vertical stack of data, with results from multiple vests from within a lot illustrated side-by-side. Right panel: Estimated *V*_50_ values.

**Fig. A1:**
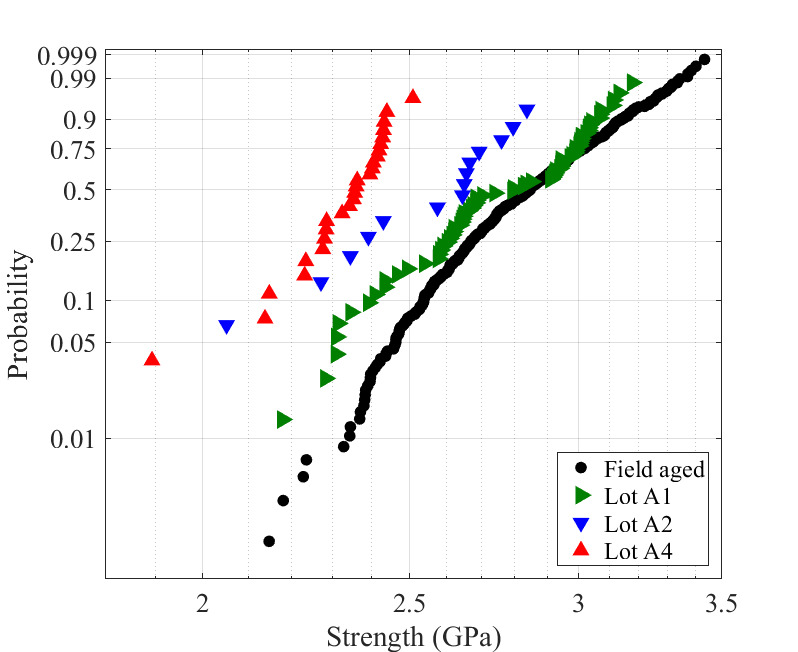

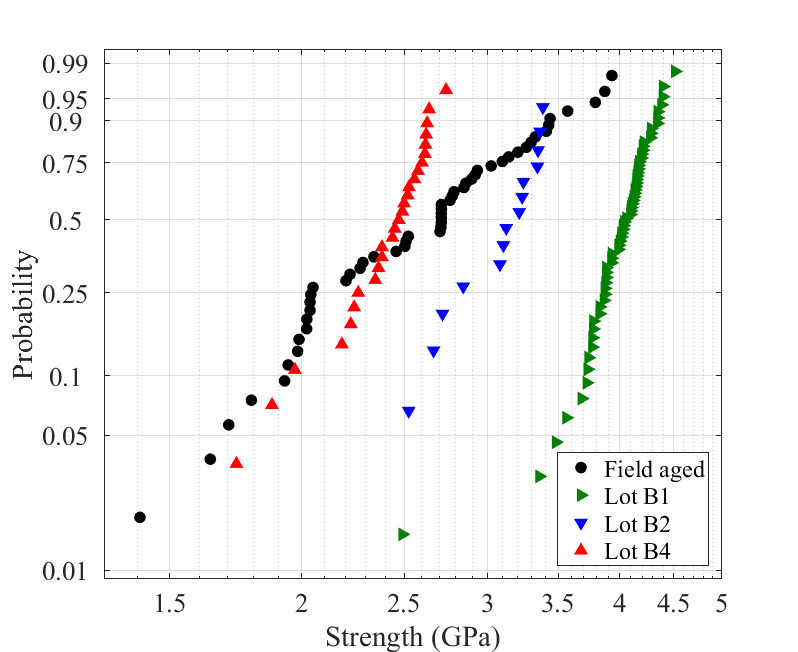
Tensile strength plotted on a Weibull probability plot, for PPTA (left) and PBO (right), comparing field-aged samples with laboratory-aged samples.

The obtained mechanical property and ballistic performance measurements are shown in Fig. 2. Mechanical properties are also shown in Fig. A1 in Weibull plot format. From the left panel of Fig. 2, we observe that the measured tensile strength values of the new, unaged PBO armor (B1) were substantially larger than those of the new, unaged PPTA armor (A1). This difference in tensile strength diminished rapidly as the armor was aged. The tensile strength of the PBO armor (B1–B4) declined significantly as a result of the artificial aging conditions, whereas the decline in tensile strength was less for the PPTA armor (A1–A4). From the right panel of Fig. 2, we observe that the aging conditions also had a considerable impact on the *V*_50_ ballistic performance for the PBO armor (B1–B3, no *V*_50_ data were collected for B4), but little to no impact on the PPTA armor (A1–A4).

**Fig. 3:**
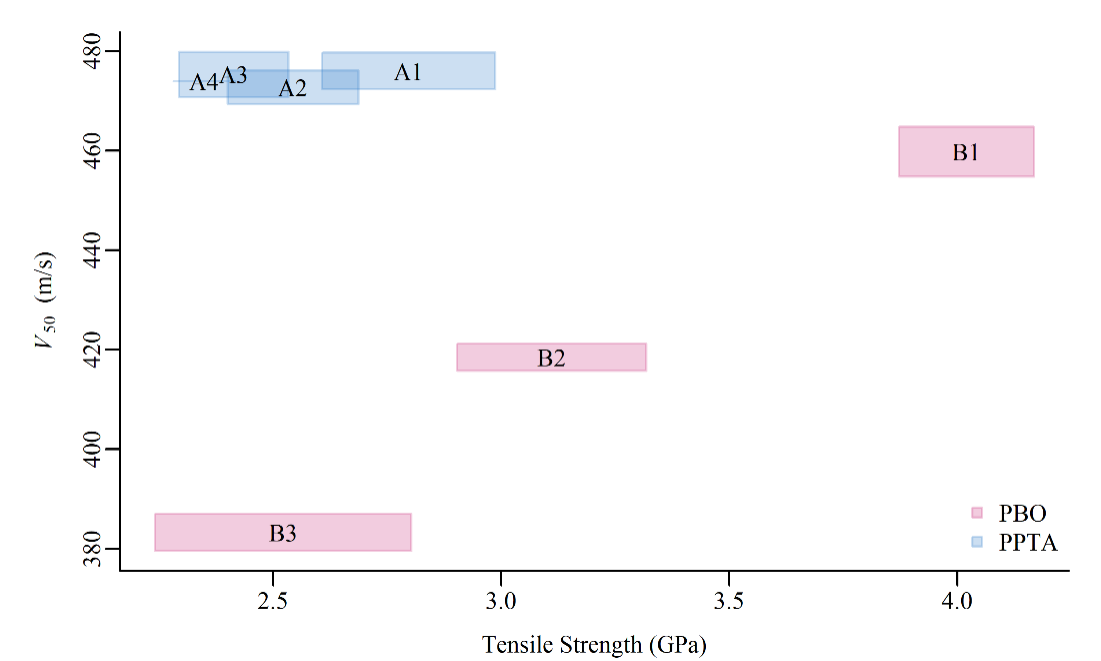
The joint interquartile range of the observed tensile strength (load at break) and ballistic performance (*V*_50_) for each armor lot.

Figure 3 displays, for each armor lot, a shaded region that represents the joint interquartile range of the observed tensile strength and ballistic performance measurements. In addition to highlighting the observations noted in the raw data in Fig. 2, Fig. 3 clearly exhibits a positive correlation between tensile strength and ballistic performance for the PBO armor (B1–B3). That is, as the tensile strength of the PBO armor increases, so too does the *V*_50_. In contrast, no such trend is observed for the PPTA armor (A1–A4). From these results, we were not able to draw inference on the impact of tensile strength on ballistic performance for PPTA armor because the aging conditions did not impact the PPTA armor in the same manner that they did the PBO armor. This observation could be attributed to a greater variability in strength for lot A1 than the other “A” lots, with a possible greater contribution from these weaker yarns on V_50_.

In summary, PBO and PPTA armor were subjected to identical artificial-aging conditions. The tensile strength of the new, unaged PBO armor was about 1.5 times greater than that of the new, unaged PPTA armor. However, the artificial aging had a severe effect on the tensile strength of the PBO armor, while only a slight effect was observed for the PPTA armor. Despite the more favorable tensile strength properties when new, the ballistic performance (*V*_50_) of the PBO armor was found to be slightly lower than that of new PPTA armor because the two types of armor were designed to withstand two different NIJ performance levels, as described in Sec. 3.1.1. Similar to the impact on tensile strength, the artificial aging also had a significant influence on the ballistic performance of the PBO armor but not on the ballistic performance of the PPTA armor.

However, it is important to note that armor performance cannot be completely captured by an analysis of material properties. Armor design, including, for example, the number of layers, their interaction with each other and the projectile, and the amount of extra material (or safety margin) built into the vest, is unique for every design, plus design considerations such as stitching and weave type can all influence ballistic results. Therefore, the conclusion that PBO armor was affected by the artificial aging and that PPTA armor was not affected is only valid for a comparison of these two particular armor designs and, in general, is not applicable to all systems containing these materials.

### 3.2 Linking Properties of Laboratory-Aged Armor to Fielded Armor

While the tensile strength and *V*_50_ results presented in the previous section indicate a link between mechanical properties of armor materials and ballistic performance, one may consider how these artificially aged laboratory materials are relevant to those observed in fielded armor, especially since the PPTA armor was relatively unchanged by the artificial aging. Accelerated testing through laboratory conditioning of armor is a convenient approach in studying the effect that degraded material properties may have on ballistic performance in fielded armor. Drawing inference from such testing requires that a link be established that illustrates that the laboratory degradation is representative of the degradation that appears in fielded armor. Therefore, we examined results from two studies of material properties (tensile strength) of fielded armor and compared them to the artificially aged armor results presented in the previous section.

#### 3.2.1 Armor Description

Tensile strength measurements from fielded PPTA armor were obtained through research efforts performed in conjunction with the Canadian government [9, 10]. The field-worn Canadian police armors were all manufactured by the same parent company and were composed entirely of PPTA yarns. The manufacturing date of the panels spanned a 10 year period from October 1992 to October 2002.

The material properties of fielded PBO armor were examined as part of Phase II of the U.S. Department of Justice’s Body Armor Safety Initiative [1–3, 5, 24]. The 75 fielded PBO-containing body armor pieces collected for this effort varied in age (17 to 71 months), material composition (15% PBO to 100% PBO), NIJ threat certification (IIA–IIIA), manufacturer, general condition, and geographic area of use.

#### 3.2.2 Determination of Extracted Yarn Mechanical Properties

Forty-one armor panels of interest from the Canadian PPTA work were selected for mechanical properties testing. These panels were selected because they were all from the same model of armor. Approximately 14 yarns were extracted from each PPTA armor panel and subjected to material properties testing using the procedure described above in Sec. 3.1.3. The tensile strength results for the 577 total PPTA yarn specimens are provided in the following section.

For the PBO armor, a total of 52 yarns were extracted from 10 fielded PBO-containing armor panels. In the cases where the armor contained materials other than PBO, only yarns containing PBO were extracted and subjected to mechanical properties testing. As with the PPTA mechanical properties testing, the PBO yarn tensile strength was measured using the procedure described above in Sec. 3.1.3. The tensile strength results for the 52 total PBO yarn specimens are provided in the following section.

#### 3.2.3 Analysis and Discussion

**Fig. 4:**
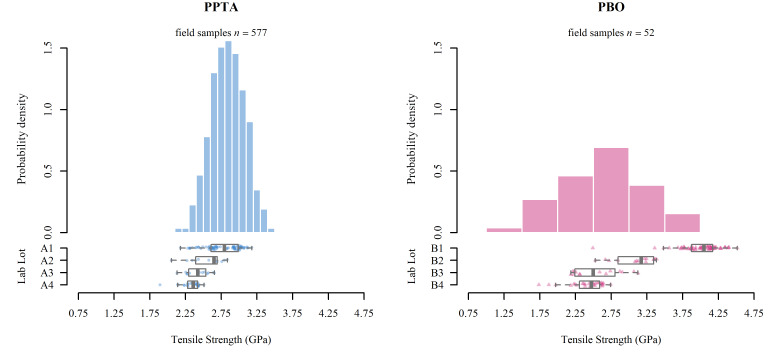
Tensile strength testing results for PPTA (left) and PBO (right) armor. The histograms illustrate the probability density functions of the tensile strength for the field-worn armor, and the box plots with individual data points provide the tensile strength of the laboratory artificially aged armor.

Figure 4 provides the distribution of the tensile strength measurements of the fibers extracted from the field-worn armor (histograms) compared to the tensile strength measurements from the laboratory artificially aged armor (box plots). The tensile strength data for both the fielded PPTA and the fielded PBO armor approximately follow a normal distribution. The tensile strength measurements for the fielded PPTA armor were tightly gathered around the mean of 2.84 GPa, with a standard deviation of 0.24 GPa, for a coefficient of variation (CV) of 8.5%. The observed minimum and maximum fielded PPTA tensile strength measurements were 2.15 GPa and 3.43 GPa, respectively. Conversely, the tensile strength measurements for the fielded PBO armor were much more spread around the mean of 2.64 GPa, with a standard deviation of 0.61 GPa, for a CV of 23.1%. The observed minimum and maximum fielded PBO tensile strength measurements were 1.41 GPa and 3.93 GPa, respectively. The observation of a larger spread of tensile strengths in PBO than in PPTA armor was also noted in the laboratory artificially aged armor. This implies that aging and environmental conditions, both field and laboratory induced, have a smaller impact on yarns extracted from PPTA armor as compared to yarns extracted from PBO armor.

In comparing the tensile strength measurements of the new, unaged PPTA armor from the laboratory aging study (Lab Lot A1) to the distribution of fielded PPTA tensile strength measurements, we observe from the left panel of Fig. 4 that the unaged laboratory armor was generally reflective of the fielded armor. While the laboratory artificial aging slightly reduced the tensile strength of the PPTA armor (Lab Lots A2–A4), the values remained consistent with the lower half of the distribution of fielded PPTA tensile strength measurements.

Conversely, when comparing the tensile strength measurements of the PBO armor from the laboratory aging study to the distribution of fielded PBO armor tensile strength measurements (right panel of Fig. 4), the tensile strength of the new, unaged PBO armor (Lab Lot B1) is reflective of only the strongest of the fielded PBO tensile strength measurements. Because the fielded PBO armor was a random sample from in-use armor, this suggests that most fielded PBO armor examined herein had experienced significant degradation in tensile strength. We observed that the laboratory artificial aging significantly reduced the tensile strength of the PBO armor (Lots B2–B4), but still, the weakest of the aged lots (B3 and B4) were reflective of the middle part of the yarn strength distribution for yarns extracted from the fielded PBO armor samples. Recall that the ballistic performance of these lots, particularly B2 and B3, was drastically lower than for the new armor lot (B1). There remains a significant proportion of the fielded PBO tensile strength distribution that exhibited lower tensile strength than even the weakest laboratory-aged lots.

In summary, while the PPTA armor only saw minor reductions in tensile strength and ballistic performance when exposed to the laboratory artificial aging conditions described in Sec. 3.1.2, the observed diminished tensile strength values were determined to be reflective of the more degraded field-worn PPTA armor. Any further reduction in tensile strength through additional artificial aging conditions would have pushed the degradation of the armor beyond what was seen in the field. This observed lack of decline in tensile strength and ballistic performance may be attributed to the robustness of the PPTA material to the aging and environmental conditions to which it was exposed. Conversely, despite the large degradation effect of laboratory artificial aging on PBO yarn strength, more aging would be required to be reflective of the lower portion of the tensile strength distribution of the fielded PBO armor. Though the observed tensile strength reduction from the artificially aged PBO armor does not conflict with that observed in the fielded armor, more aggressive laboratory aging would be required to fully reflect the tensile strength loss in extracted yarn samples from the most degraded field-worn PBO armor.

In this section, we have presented studies that demonstrate the effect of degraded material properties on ballistic performance in armor, and we have linked the degradation observed in the laboratory artificially aged armor to that found in fielded armor. Next, we used these data to demonstrate how the theoretical degradation relationships developed in Sec. 2 may be applied to predict ballistic performance from more easily measured mechanical properties.

## 4 Validation Results: Predicting Armor Performance Using Dimensional Analysis

As previously discussed, dimensional analysis relating the properties of fiber strain-wave speed and specific toughness [14–19] can be used to predict a theoretical *V*_50_. This concept could be a powerful tool in predicting the effect of a change in fiber properties (such as changes in tensile strength, elongation at break, or modulus due to aging) on the ballistic performance of an armor system. This theoretical *V*_50_ could then be used to determine when a change in the material properties measured from an armor sample might translate into a loss in performance in the case of field and laboratory aging studies, or an increase in performance in the case of new or improved fibers.

Sometimes, only very limited data or samples are available from a fielded or laboratory-aged armor, and it is not possible to conduct extensive *V*_50_ testing. In order to develop a *V*_50_ with reasonable confidence, multiple panels should be tested, but large sample sizes might not be available, particularly ones that have been subjected to identical environments and service in the field. In these cases, where multiple yarns can be extracted from a single armor sample, a theoretically determined *V*_50_ could potentially be used to determine some point, perhaps a conservative one, at which measured degradation in mechanical properties might translate into a critical reduction in ballistic performance. However, the relationship between the theoretically predicted performance and actual measured performance must be understood before the value of this approach can be realized.

Regarding such theoretical estimations of *V*_50_ from yarn strength data, it is critical to emphasize that the purpose is not to determine absolute values of *V*_50_, for any particular armor, but instead, the purpose is to determine relative *V*_50_ behavior in ratio form, whereby the influence of degradation in mechanical material properties of the key armor materials on its ballistic performance is assessed, all other things being equal.

**Fig. 5:**
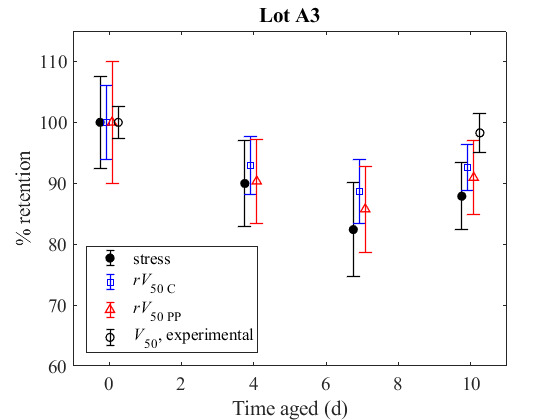

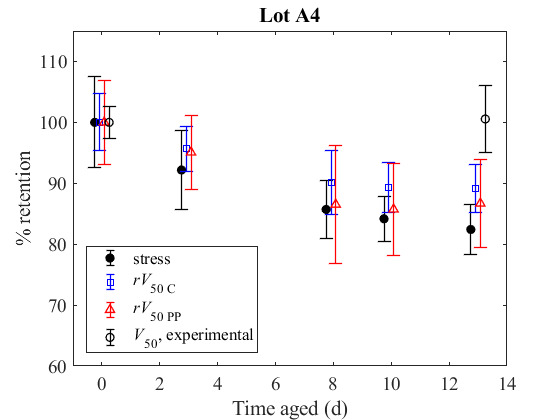
Tensile strength retention and *V*_50_ retention, relative to the initial, unaged values, as computed using both the Cunniff (C) model and the Phoenix-Porwal (PP) model. Predicted and measured *V*_50_ values are shown for PPTA armor (a) Lot A3 and (b) Lot A4 (see Table 3 for environmental conditioning). Error bars represent the 95% confidence interval for the scale parameter from a logistic fit of the experimental *V*_50_ data, and standard deviation of an average of at least 10 measurements, scaled by the initial mean, for the other lines. Values in (a) and (b) are tabulated in Table A1 and Table A2, respectively.

**Fig. 6:**
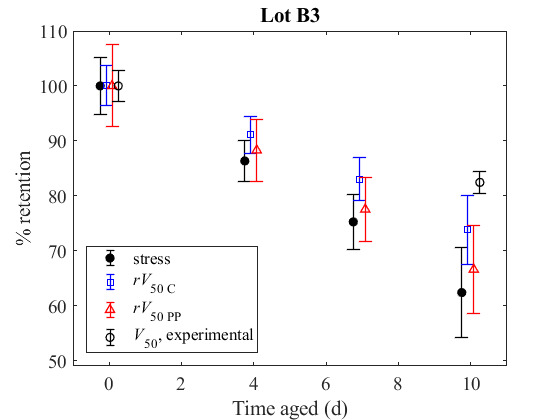

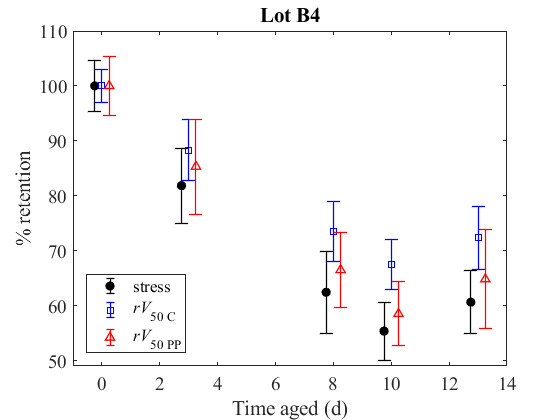
Tensile strength retention, predicted *V*_50_ retention, and measured *V*_50_ reduction relative to the initial, unaged values for PBO armor (a) Lot B3 and (b) Lot B4 (see Table 3 for environmental conditioning). Error bars represent the 95% confidence interval for the scale parameter from a logistic fit of the experimental *V*_50_ data, and standard deviation of an average of at least 10 measurements, scaled by the initial mean, for the other lines. Values in (a) and (b) are tabulated in Table A3 and Table A4, respectively.

To examine this relationship, the predicted *V*_50_ based on material properties measured from yarn specimens from the aged armor was compared with actual measured ballistic limit results from armor exposed in the same manner, using the *V*_50_ of the aged armor divided by the original *V*_50_, the ratio called *V*_50_ retention. Figures 5 and 6 show, in ratio form, yarn tensile strength retention, predicted *V*_50_ retention, using both the Cunniff and Phoenix-Porwal methods from Eq. (7) and Eq. (12), respectively, and last, actual *V*_50_ retention for aged samples. For the Phoenix-Porwal model, $\psi_{\text{max}}$ was fixed at 2.87. Tables A1 through A4, in the Appendix, give ratio values for tensile strength retention, failure strain retention, and predicted *V*_50_ retention using both the Cunniff model and the Phoenix-Porwal model. Large error bars on the Phoenix-Porwal’s *V*_50_ retention typically are due to uncertainty in strain values, as can be seen in the table associated with the figure. The influence of the strain on Phoenix-Porwal’s *V*_50_ retention is also why it does not always follow the same trend as the tensile strength and Cunniff’s *V*_50_ retention. Cunniff’s *V*_50_ retention is influenced much less by the failure strain and thus typically matches the trends of the failure stress.

Yarns extracted from the PPTA armor from Lot A4 exhibited a decline in tensile strength of approximately 18%, and the armor from Lot A3 exhibited a decline of approximately 11%. In both cases, the actual measured *V*_50_ of the PPTA armor was relatively unchanged by the conditioning protocols. The predicted *V*_50_ showed a theoretical decline of approximately 11% and 13% for the Cunniff model and the Phoenix-Porwal model, respectively, for Lot A4, and a theoretical decline of approximately 7% and 9% for the Cunniff model and the Phoenix-Porwal model, respectively, for Lot A3. It is interesting to note that the predicted *V*_50_ consistently provided a conservative estimate of the *V*_50_ of the degraded PPTA system, and that the predicted ratio appears to be more sensitive to changes in tensile strength for this system than the actual measured *V*_50_. This may be attributed to the differences in material behavior at the slow, quasi-static rates where the tensile testing is performed, as compared to the extremely fast time scale of the ballistic tests. In addition, changes such as material susceptibility to mechanical damage and armor design are not considered in these models. Furthermore, the Phoenix-Porwal model is more conservative than the Cunniff model, which is consistent with the factor in Eq. (13) being less than unity in the case where the failure strain after aging is less than the failure strain before aging. These results demonstrate that the PPTA system examined here is robust and can withstand some yarn material degradation before changes in experimentally determined *V*_50_ are observed.

This analysis was also applied to similar data sets for the PBO armor. Figure 6 shows tensile strength retention, predicted *V*_50_ retention, and measured *V*_50_ retention for aged PBO samples. Yarns extracted from the PBO armor from Lot B4 exhibited an approximate reduction in tensile strength of 40% after exposure, and the armor from Lot B3 exhibited a reduction in tensile strength of approximately 38% after exposure. In both cases, the actual measured *V*_50_ of the PBO armor was reduced by 15% to 18% by the aging. The predicted *V*_50_ showed a theoretical decline of 28% and 35% for the Cunniff model and the Phoenix-Porwal models, respectively. For Lot B4, the corresponding *V*_50_ declines were 26% and 33% for the Cunniff and the Phoenix-Porwal models, respectively, for Lot B3.

In summary, the theoretical predictions for *V*_50_ follow the same trends as the tensile properties with aging and provide conservative estimates for the measured *V*_50_ for both the PPTA and PBO armors. The two different theoretical methods predict similar results, differing by less than 5% in predicted *V*_50_ reduction.

## 5 Conclusions and Future Work

In conclusion, there appears to be a correlation between changes in mechanical properties and changes in ballistic performance for PBO armor exposed to different aging conditions. This relationship is not observed for PPTA armor when aged under the same conditions. Theoretical *V*_50_ values were calculated for new and aged PPTA and PBO armor systems and compared to the actual change in ballistic performance. Both Cunniff’s and Phoenix-and-Porwal’s equations for theoretical *V*_50_ appeared to provide a conservative estimate of the change in ballistic performance for both PPTA and PBO armor.

This work raises the possibility of an opportunity to include an “armor witness coupon sample” made up of extra material from the armor and inserted into the ballistic panel, where it could be easily sampled. Material properties from this coupon sample when new could be cataloged in a database for future reference. This coupon sample could then be removed from the armor panel and its material properties analyzed later in the vest’s service lifetime. Then, *V*_50_ could be computed and compared to the original material parameters. This approach could serve as a useful tool for fielded armor performance surveillance programs relying upon testing of armor coupon samples. While this study has provided some information, more analyses of field-aged and laboratory-aged armor systems are critical to fully understand and predict armor service life, especially as commonly used fibers are improved and new fibers and technologies are introduced into the marketplace.

## 6 Appendix A

In the tables below, the mean is denoted by μ; the standard deviation is denoted by σ; the normalized mean is denoted by $\mu_{n}$, where$\mu_{n}=\frac{\mu_{t}}{\mu_{0}}$, and where $\mu_{0}$ is the mean at day 0, and the coefficient of variation is denoted by CV, where $CV=\frac{\mu }{\sigma }$.

**Table A1:** Tensile stress retention, *V*_50_ retention relative to the initial, unaged values, as computed using both the Cunniff model and the Phoenix-Porwal (P & P) model, and measured *V*_50_ for PPTA armor from Lot A3. Values with a subscript ‘n’ are normalized by the initial mean on day 0. Values are the average of at least 13 tensile tests.

	Stress				Strain				Cunniff		P & P	
Day	*µ* (GPa)	σ (GPa)	$\mu_{n}$ (%)	*CV*	*µ* (%)	σ (%)	$\mu_{n}$ (%)	*CV*	$\mu_{n}$ (%)	*CV*	$\mu_{n}$ (%)	*CV*
0	2.76	0.209	1.000	13.2	2.68	0.996	1.000	2.69	1.000	16.5	1.000	9.95
4	2.48	0.194	0.900	12.8	2.59	0.318	0.966	8.15	0.930	19.6	0.903	13.2
7	2.27	0.212	0.824	10.7	2.54	0.281	0.947	9.04	0.887	17.1	0.858	12.1
10	2.42	0.152	0.879	15.9	2.71	0.342	1.009	7.92	0.926	24.4	0.909	15.1

**Table A2:** Tensile stress retention, *V*_50_ retention using both the Cunniff model and the Phoenix-Porwal (P & P) model, and measured *V*_50_ for PPTA armor from Lot A4. Values with a subscript ‘n’ are normalized by the initial mean on day 0. Values are an average from two different samples, each consisting of at least 13 tensile tests.

	Stress				Strain				Cunniff		P & P	
Day	*µ* (GPa)	σ (GPa)	$\mu_{n}$ (%)	*CV*	*µ* (%)	σ (%)	$\mu_{n}$ (%)	*CV*	$\mu_{n}$ (%)	*CV*	$\mu_{n}$ (%)	*CV*
0	2.84	0.212	1.000	13.4	2.99	0.340	1.000	8.79	1.000	21.4	1.000	14.5
3	2.62	0.183	0.922	14.3	2.94	0.454	0.982	6.47	0.956	26.3	0.951	15.6
8	2.43	0.133	0.857	18.3	2.56	0.570	0.857	4.50	0.901	17.2	0.865	8.96
10	2.39	0.105	0.842	22.7	2.55	0.425	0.853	6.00	0.893	21.6	0.857	11.3
13	2.34	0.117	0.824	19.9	2.69	0.459	0.899	5.86	0.891	22.5	0.867	11.9

**Table A3:** Tensile stress retention, *V*_50_ retention using both the Cunniff model and the Phoenix-Porwal (P & P) model, and measured *V*_50_ reduction relative to the initial, unaged values for PBO from Lot B3. Values with a subscript ‘n’ are normalized by the initial mean on day 0. Values are the average of at least 13 tensile tests.

	Stress				Strain				Cunniff		P & P	
Day	*µ* (GPa)	σ (GPa)	$\mu_{n}$ (%)	*CV*	*µ* (%)	σ (%)	$\mu_{n}$ (%)	*CV*	$\mu_{n}$ (%)	*CV*	$\mu_{n}$ (%)	*CV*
0	4.08	0.213	1.000	19.1	2.59	0.404	1.000	6.42	1.000	27.4	1.000	13.4
4	3.52	0.153	0.863	23.0	2.28	0.260	0.880	8.76	0.911	27.1	0.883	15.6
7	3.07	0.204	0.752	15.1	1.98	0.258	0.763	7.68	0.830	21.2	0.775	13.1
10	2.55	0.332	0.624	7.7	1.73	0.275	0.665	6.270	0.738	11.8	0.666	8.32

**Table A4:** Tensile stress retention, *V*_50_ retention using both the Cunniff model and the Phoenix-Porwal (P & P) model, and measured *V*_50_ reduction relative to the initial, unaged values for PBO from Lot B4. Values with a subscript ‘n’ are normalized by the initial mean on day 0. Values are an average from two different samples, each consisting of at least 13 tensile tests.

	Stress				Strain				Cunniff		P & P	
Day	*µ* (GPa)	σ (GPa)	$\mu_{n}$ (%)	*CV*	*µ* (%)	σ (%)	$\mu_{n}$ (%)	*CV*	$\mu_{n}$ (%)	*CV*	$\mu_{n}$ (%)	*CV*
0	3.96	0.186	1.000	21.3	2.79	0.333	1.000	8.36	1.000	34.0	1.000	18.6
3	3.24	0.270	0.818	12.0	2.43	0.397	0.871	6.11	0.883	15.8	0.853	9.86
8	2.47	0.294	0.624	8.39	1.86	0.286	0.669	6.51	0.736	13.5	0.665	9.71
10	2.19	0.209	0.553	10.5	1.59	0.245	0.569	6.48	0.675	15.1	0.585	10.0
13	2.40	0.229	0.606	10.5	1.82	0.445	0.653	4.09	0.723	12.6	0.648	7.23
